# Hypovitaminosis D: Is It Time to Consider the Use of Calcifediol?

**DOI:** 10.3390/nu11051016

**Published:** 2019-05-06

**Authors:** Roberto Cesareo, Alberto Falchetti, Roberto Attanasio, Gaia Tabacco, Anda Mihaela Naciu, Andrea Palermo

**Affiliations:** 1 Unit of Metabolic Diseases, Department of Internal Medicine, S. Maria Goretti Hospital, 04100 Latina, Italy; 2 Istituto Auxologico Italiano, IRCCS, Unit for Bone Metabolism Diseases and Diabetes & Lab of Endocrine and Metabolic Research; University of Milan, and EndOsMet, Villa Donatello Private Hospital, 50100 Florence, Italy; alberto.falchetti2@alice.it; 3 Endocrinology Service, IRCCS Orthopedic Institute Galeazzi, 20161 Milan, Italy; roberto.serena@libero.it; 4 Unit of Endocrinology and Diabetes, University Campus Bio-Medico, 00128 Rome, Italy; g.tabacco@unicampus.it (G.T.); a.naciu@unicampus.it (A.M.N.); a.palermo@unicampus.it (A.P.)

**Keywords:** vitamin D, cholecalciferol, calcifediol, hypovitaminosis D

## Abstract

Hypovitaminosis D is becoming a notable health problem worldwide. A consensus exists among several different medical societies as to the need for adequate levels of vitamin D for bone and general health. The correct method by which to restore normal vitamin D levels is still a matter of debate. Although cholecalciferol remains the most commonly distributed form of vitamin D supplementation worldwide, several drugs with vitamin D activity are available for clinical use, and making the correct selection for the individual patient may be challenging. In this narrative review, we aim to contribute to the current knowledge base on the possible and appropriate use of calcifediol—the 25-alpha-hydroxylated metabolite—in relation to its chemical characteristics, its biological properties, and its pathophysiological aspects. Furthermore, we examine the trials that have aimed to evaluate the effect of calcifediol on the restoration of normal vitamin D levels. Calcifediol is more soluble than cholecalciferol in organic solvents, due to its high polarity. Good intestinal absorption and high affinity for the vitamin-D-binding protein positively affect the bioavailability of calcifediol compared with cholecalciferol. In particular, orally administered calcifediol shows a much shorter half-life than oral cholecalciferol. Most findings suggest that oral calcifediol is about three- to five-fold more powerful than oral cholecalciferol, and that it has a higher rate of intestinal absorption. Accordingly, calcifediol can be particularly useful in treating diseases associated with decreased intestinal absorption, as well as obesity (given its lower trapping in the adipose tissue) and potentially neurological diseases treated with drugs that interfere with the hepatic cytochrome P-450 enzyme system, resulting in decreased synthesis of calcifediol. Up to now, there has not been enough clinical evidence for its use in the context of osteoporosis treatment.

## 1. Introduction

The prevalence of hypovitaminosis D indicates that it is a common problem worldwide, as identified in numerous epidemiological studies [1]. Opinions regarding the optimal concentration of serum 25-hydroxyvitamin D (25(OH)D) vary widely among institutions and scientific societies. Vitamin D deficiency has historically been defined as circulating levels of 25(OH)D lower than 20 ng/mL. According to the Institute of Medicine (IOM), the recommended levels of at least 20 ng/mL (50 nmol/L) meet the needs of at least 97.5% of the global population [2], whereas other authors have defined vitamin D insufficiency as a 25(OH)D concentration of 21–29 ng/mL, and declared the optimal level of 25(OH)D to be more than 30 ng/mL (75 nmol/L) [3].

Vitamin D supplements are the most widely used strategy to restore vitamin status. By diagnosing subjects with 25(OH)D levels between 20 and 29 ng/mL as deficient, the costs of vitamin D supplementation are liable to become increasingly higher. Although human studies have strongly suggested a link between vitamin D deficiency and impaired bone health [4], the results of intervention studies have not yet convincingly shown positive extra-skeletal effects of vitamin D [5]. However, several therapeutic strategies have been proposed to deal with vitamin D deficiency, and, up to now, cholecalciferol seems to be the most used supplement.

Recently, Quesada-Gomez and colleague reviewed all studies dealing with a comparison of cholecalciferol with calcifediol, showing that oral calcifediol results in more potent action and rapid increase in serum 25(OH)D compared to cholecalciferol [6].

In this narrative review, we aim to contribute to the current knowledge base on the possible and appropriate clinical use of calcifediol—the 25-alpha-hydroxylated metabolite—in relation to its chemical characteristics, its biological properties, and its pathophysiological aspects. The content of this review, which includes a description of potential advantages and limitations, provides insight into whether this form of vitamin D supplementation can be effectively prescribed for the treatment of human conditions and pathologies and/or individuals.

The more informed use and prescription of both cholecalciferol and calcifediol could confer considerable health benefits, with the potential consequence of better economic resource allocation. A desirable benefit–cost ratio could also be reached if subjects at higher risk for vitamin D deficiency were to be appropriately treated.

### 1.1. Biosynthesis of Vitamin D

Vitamin D or cholecalciferol, also termed D_3_, represents the natural form of vitamin D. It is produced in skin exposed to sunlight from 7-dehydrocholesterol (7-DHC), and is present in only a few foods, mainly including fortified dairy products and fish oils. The precursor, 7-DHC, produces pre-vitamin D, successively undergoing a temperature-sensitive rearrangement of three double bonds to form vitamin D. Synthesis in the skin is thus the most important source of vitamin D and is based on the intensity of ultraviolet irradiation, which is dependent on both the seasonal period and latitude [7,8]. Once in the form of D_3_, it is transported to the liver through the blood while bound to either albumin or vitamin-D-binding protein (DBP) [9]. Cholecalciferol then undergoes one of three hydroxylation steps to produce either active or inactive metabolites. Specifically, the three possible reactions are (1) liver 25-hydroxylation, with the intermediate production of 25-hydroxyvitamin D, 25(OH)D or calcifediol, also known as calcidiol; (2) 1α-hydroxylation, which leads to the final production of 1,25(OH)_2_D or calcitriol; and (3) 24-hydroxylation, which produces either the inactive 24,25(OH)_2_D or calcitroic acid, which is excreted in bile, and 1,24,25(OH)_3_D metabolites. However, 24,25(OH)_2_D may contribute to 25(OH)D measurements; this is a possible problem, as its presence in the serum can be considered both a nuisance and a nutritionally valuable molecule [10].

The 25(OH)D molecule is the major circulating form of vitamin D, although its synthesis has not been reported to be highly regulated [11]. As vitamin D_3_ is highly lipophilic, supplementation of high amounts may saturate the adipose tissue. Afterward, it is readily converted to 25(OH)D [9], which might be responsible for vitamin D toxicity, since there are no known regulatory mechanisms for this conversion to 25(OH)D [9,12].

### 1.2. Vitamin-D-Binding Protein (DBP)

DBP is a multitasking alpha-globulin with roles in (1) transportation and storage of vitamin D, (2) actin scavenging, (3) macrophage-activating factor precursor, (4) fatty acid binding, and (5) C5a-mediated chemotaxis [13].

In hyperestrogenism, which occurs during pregnancy, DBP levels increase by up to 50%. This is also the case in premenopausal women undergoing estrogenic treatments, such as oral hormonal contraceptive intake, due to which their total vitamin D values increase. Postmenopausal women, who have very low circulating estrogen levels, have the lowest DBP concentrations and hence the lowest total vitamin D levels. In severe hepatic failure, DBP decreases [14].

Affinity for DBP is the major determinant of the length of time a vitamin D metabolite remains in circulation. Vitamin D, 25(OH)D, and 1,25(OH)_2_D have substantially different dissociation constants: approximately 10^−9^ for 25(OH)D and approximately 10^−7^ for vitamin D and 1,25(OH)_2_D [15]. The dissociation constant regulates the “free” vitamin D concentration for diffusion through the cell membrane and metabolism with the modulation of cell activity. Such different dissociation constants contribute to the different circulating half-lives of the metabolites: vitamin D, stored in the adipose tissues of the body, has a half-life of approximately two days; 25(OH)D has a half-life of three weeks; and 1,25(OH)_2_D has a half-life of only a few hours [9,16]. Thus, DBP maintains stable concentrations of vitamin D metabolites and modulates the speed of their bioavailability, their activation, and the reactivity of the final target organ [17].

The gene for DBP (GC) is exceptionally polymorphic [18], and the distribution of its genetic variants depends on country and ethnicity. Different levels of 25(OH)D could be the result of differences in the affinity between DBP/GC phenotypes and 25(OH)D [19].

Besides GC polymorphism, DNA variants in at least three other genes have been shown to influence the 25(OH)D serum levels, namely, 7-DHC reductase, CYP2R1 25-hydroxylase, and CYP24A1 24-hydroxylase. However, the overall impact of these four genes on the changes in 25(OH)D serum levels is only about 5% [20].

### 1.3. Cytochrome P-450 Enzymes (CYPs) with 25-Hydroxylase Activity

All the above-mentioned enzymatic activities occur by CYP mixed-function oxidases. This is a family of heme-containing enzymes that absorb light at 450 nm in their reduced states. The liver represents the major source of 25(OH)D production from vitamin D. CYP2R1, first identified as a microsomal vitamin D 25-hydroxylase, is considered to be the key vitamin D 25-hydroxylase [21]. CYP2R1, primarily expressed in the liver and testes, 25-hydroxylates both D_2_ and D_3_ with comparable kinetics. Although CYP2R1 represents the principal human vitamin D 25-hydroxylase, its deficiency is extremely rare. However, Roizen et al. recently showed in their in vivo animal study that the expression of CYP2R1 is significantly reduced in obesity; this finding, in addition to the sequestration of highly lipophilic cholecalciferol by adipose tissue, might justify the decreased circulating 25(OH)D [22]. Other conditions can affect CYP2R1 expression and activity. In particular, Aatsinki and colleagues clearly showed that both fasting and diabetes suppressed hepatic murine CYP2R1 via overexpression of coactivator peroxisome proliferator-activated receptor gamma coactivator 1-α and glucocorticoid receptor activation [23].

The circulating level of 25(OH)D can be regarded as a useful marker of a subject’s vitamin D status—a result of dietary intake of vitamin D and exposure to ultraviolet radiation.

Drugs such as ketoconazole and fluconazole or phenobarbital inhibit the synthesis of both 25(OH)D and 1,25-OH_2_D by directly targeting the key conversion enzymes [24,25].

### 1.4. Diseases/Conditions Associated with Mutations/Variants of CYP Genes

Several genome-wide association studies have implicated genetics as a factor that contributes to the circulating levels of 25(OH)D. Among the relatively scarce number of genes associated with serum levels of 25(OH)D is *CYP2R1* [26,27,28]. More specifically, patients with a mutation in the *CYP2R1* gene have a 25(OH)D deficiency and symptoms of vitamin-D-dependent rickets, particularly vitamin-D-dependent rickets type 1B (VDDR1B; MIM 600081), a very rare condition reported to affect only a few families of patients [29]. Consequently, this context represents the ideal indication for 25-hydroxylated vitamin D metabolite use.

Several studies have revealed that polymorphisms in or near the *CYP2R1* gene are associated with the circulating concentrations of 25(OH)D [27,29,30,31,32,33], and they may also be associated with modified responses to oral vitamin D supplementation [34], possibly by influencing DNA methylation [35].

## 2. Materials and Methods

**Figure 1:**
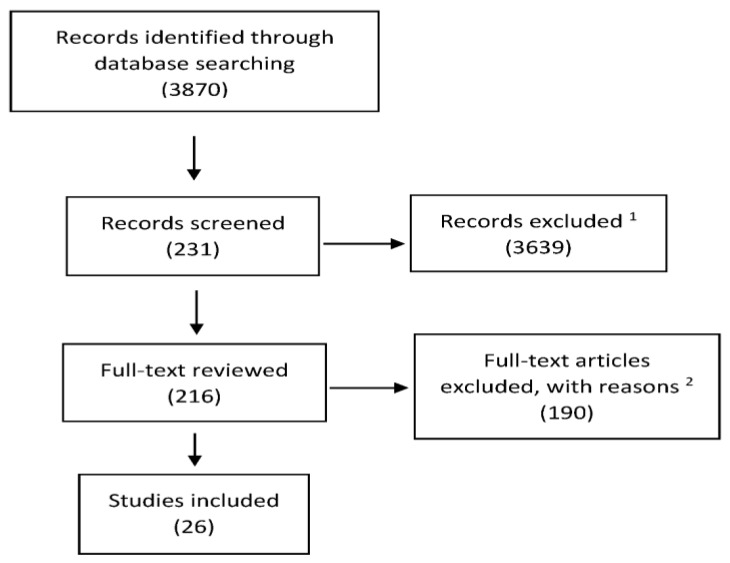
Study selection process. ^1^ Exclusion criteria included non-human subjects, non-primary research, lack of a primary outcome related to hypovitaminosis D and/or bone health and/or secondary hyperparathyroidism, abstract-only publication, or non-English language. ^2^ Additional exclusion criteria for full texts included a pediatric population, diabetic population, case reports, and failure to measure baseline and post-treatment 25(OH)D levels. We also excluded papers focused on cardiovascular diseases and overall chronic human disease.

PubMed and MEDLINE were searched in conformance with PRISMA guidelines [36,37] to identify publications about the efficacy of calcifediol supplementation in subjects with hypovitaminosis D and other particular conditions. Specifically, we considered studies that examined the potential relationship between calcifediol and 25(OH)D levels and bone metabolism, secondary hyperparathyroidism (SHPT), muscle function and risk of falls, fragility fractures, malabsorption, and obesity. We also considered the direct comparison between calcifediol and cholecalciferol. The following search terms were used: calcifediol, 25(OH)D, vitamin D, cholecalciferol, vitamin D concentrations, hypovitaminosis D, bone, hyperparathyroidism, anticonvulsant, calcium absorption, osteoporosis, fractures, falls, fracture risk, pharmacokinetics, malabsorption, obesity, glucocorticoids, antineoplastics, and antiretrovirals. The search strategy was conducted in the following databases: PubMed, Embase, Cochrane Database of Systematic Reviews, and selected gray literature sources. The date range was from the inception of the respective database until January 2019. Eligibility criteria for inclusion in the final review were: only human studies and interventional studies. Only publications in English were included. The study selection process is illustrated in Figure 1.

## 3. Vitamin D and Pathophysiological Related Effects on Calcium Absorption, Secondary Hyperparathyroidism, and Bone

Through a vitamin D receptor (VDR)-mediated interaction, vitamin D operates in Ca^2+^ transportation tissues, such as intestine and kidney, Ca^2+^-sensitive parathyroid tissue, and bone homeostasis.

### 3.1. Calcium Absorption

Vitamin D is essential for normal intestinal calcium absorption, but it is not yet clear what 25(OH)D level is optimal for calcium absorption. In a clinical study, the authors concluded that vitamin D deficiency did not decrease serum 1,25(OH)_2_ D, and therefore calcium absorption, until serum 25(OH)D fell to approximately 4 ng/mL [38]. The Endocrine Society (ES) guidelines suggest that calcium absorption plateaus at a serum 25(OH)D level of 30 ng/mL [3]. To support these conclusions, the authors quoted a paper showing that intestinal calcium absorption in postmenopausal women increased by 45–65% when their mean blood level of 25(OH)D increased from 20 to 32 ng/mL [39]. In another randomized, double-blind, placebo-controlled trial in postmenopausal women, daily vitamin D doses between 400 and 4800 IU were administered for one year. Even though serum 25(OH)D increased to 66 ng/mL, the increase in absorbed calcium was only 6% with the highest dose. Malabsorption occurred only when serum 25(OH)D was very low, as in severe vitamin D deficiency, defined as a serum 25(OH)D level below 10 ng/mL, with a reduction in 1,25(OH)_2_D production due to the lack of substrate [40]. Finally, more recent studies have failed to show evidence of a threshold for calcium absorption, with serum 25(OH)D levels ranging from 16 to 52 ng/mL. According to these authors, calcium absorption is not a useful biomarker for determining nutritional recommendations for vitamin D within that range [41].

### 3.2. Secondary Hyperparathyroidism (SHPT)

Various studies have mentioned the inverse correlation between PTH levels and 25(OH)D serum concentration. Further decreases in PTH levels did not occur in subjects whose 25(OH)D levels were between 30 and 40 ng/mL [1,42,43]. Augmented osteoclastic activity, which is mediated by PTH, produces local foci of bone weakness and determines a generalized decline in bone mineral density (BMD), resulting in osteopenia and/or osteoporosis. In this context, when the patients are affected only by vitamin D insufficiency, the initial elevation of PTH levels is clearly different from the defined picture of SHPT that is observed mainly when 25(OH)D levels are markedly lower than 20 ng/mL [43]. Regarding nutritional rickets, laboratory observations have demonstrated that PTH levels increase when 25(OH)D levels drop below 13.6 ng/mL. Taken together, the evidence points to 12–13.5 ng/mL as the critical cut-off 25(OH)D level below which nutritional rickets is more likely to occur [44,45].

In a cross-sectional study [46], SHPT was found in about 34% of subjects whose serum 25(OH)D level was below 12 ng/mL, and the authors underlined that, even at that level, there was no evidence of SHPT in the majority of individuals. No biochemical evidence was thus found in support of designating an insufficiency state for a serum 25(OH)D concentration between 12 and 30 ng/mL.

Many conflicting results may be due to differing availabilities of assays and methods used for assessing the levels of both the serum 25(OH)D and PTH, as well as to the likely varied daily calcium intake existing among the various populations considered in the studies.

Several randomized clinical trials (RCT) would seem to define 20 ng/mL as the cut-off 25(OH)D level below which serum PTH starts to increase in vitamin D deficient subjects [47,48,49]. Finally, a systematic review and meta-analysis of 18 RCTs has clearly shown that 1000 IU vitamin D daily supplementation can suppress serum PTH levels even in overweight/obese adults [50]. In our opinion, future research is needed to determine the exact threshold below which higher serum levels of PTH could determine clinically meaningful outcomes in several diseases.

### 3.3. Bone, Osteomalacia, Osteoporosis, and Fractures

The diagnosis of osteomalacia can be confirmed by an iliac crest bone biopsy, although this is rarely performed because of its cost, patient discomfort, and limited availability of the procedure. Priemel et al. [51] examined 675 iliac crest cadaveric biopsies to evaluate structural histomorphometric parameters and osteoid indices. The authors did not find a pathological accumulation of osteoid in any subjects whose serum 25(OH)D was higher than 30 ng/mL. Large osteoid areas were shown mostly in patients with 25(OH)D levels <20 ng/mL or, less frequently, between 20 and 30 ng/mL. The investigators concluded that to preserve skeletal health, vitamin D supplementation along with an adequate calcium intake should achieve circulating levels of 25(OH)D higher than 30 ng/mL. However, as acknowledged by the authors, the study had several biases: the gold standard for the quantification of bone formation, i.e., tetracycline double labeling, could obviously not be applied, and other data were missing as well, e.g., calcium and PTH levels, and kidney function [51].

For osteoporosis, the gold standard for showing the efficacy of its treatment is a reduced fracture risk, but the correlation between hypovitaminosis D, osteoporosis, and fractures and treatment with vitamin D is quite poor.

Regarding BMD, a clinical study showed that a significant reduction in bone resorption occurred in men and women receiving daily supplements of 400–1000 IU vitamin D [3]. A randomized, double-blind, and controlled clinical study was conducted to evaluate the efficacy of vitamin D in postmenopausal women with basal 25(OH)D levels ranging from 14 to 27 ng/mL [52]. It showed a trivial effect on calcium absorption and no clinical benefit on BMD, muscle function, and falls. The authors thus supported the IOM position that vitamin D deposits are replete in subjects whose serum 25(OH)D level is higher than 20 ng/mL.

In contrast to the previous reports about the efficacy of vitamin D in preventing fractures, a Cochrane review including 45 clinical trials concluded that vitamin D alone appears unlikely to be effective in preventing hip fractures, vertebral fractures, or any new fractures [53,54,55,56]. The authors of a large metanalysis demonstrated that vitamin D supplementation does not have a clinically relevant impact on fractures, falls, and BMD, a conclusion that is unlikely to change with future similar studies. The investigators stated that the evidence supporting the use of vitamin D supplements for the preservation or improvement of musculoskeletal health is poor—an aspect to be included in clinical guidelines. The main exception is the prevention or treatment of rickets and osteomalacia, conditions that may occur after long periods without sun exposure, bringing 25(OH)D levels below 10 ng/mL [57].

## 4. Pharmacokinetics of Calcifediol

### 4.1. General Remarks

Vitamin D_2_ (ergocalciferol) and D_3_ (cholecalciferol) are still the most common forms of vitamin D supplementation. However, supplementation with calcifediol, the 25-hydroxylated metabolite, might be considered to be equally effective as a supplementation strategy in cases of malabsorption or impaired hepatic function.

Contributing to vitamin D status are several known factors, such as sun exposure, skin color, body weight, and food-related vitamin D intake; however, they account for less than 50% of the observed variance in circulating levels of 25(OH)D. While cholecalciferol supplementation is not associated with a marked reduction in inter-individual variation, oral calcifediol supplements could potentially reduce such inter-individual variation, since it reduces the number of steps involved in both the absorption and metabolism of vitamin D into 25(OH)D. In fact, calcifediol is more hydrophilic and already 25-hydroxylated [58].

The intestinal absorption of calcifediol has been demonstrated to have an efficacy higher than that of cholecalciferol. Calcifediol is absorbed through the portal vein circulation, whereas more complex cholecalciferol uptake occurs through the lymphatic pathway. Such differences in transportation may partially contribute to the greater overall bioavailability of calcifediol [59].

When baseline 25(OH)D levels are very low, there is an estimated average increase of 0.7–1 ng/mL for every 100 IU of daily ingested vitamin D. Subsequently, the increase slows as 25(OH)D levels rise, indicating that cholecalciferol absorption is not linear [60]. Conversely, a multicentric interventional study in postmenopausal women suggested that, as opposed to cholecalciferol, calcifediol is characterized by linear absorption when administered in either a daily or weekly regimen. Over a three month period of supplementation, 25(OH)D levels were successfully raised without altering other markers related to bone and mineral metabolism. This also suggests that a safe and effective short- and mid-term effect on mineral metabolism occurs with both daily and weekly dosages of calcifediol when used to treat vitamin D inadequacy or deficiency in this population of subjects [61]. Thus, a potential additional advantage of calcifediol is that the increase in 25(OH)D is linear and independent of the baseline values of 25(OH)D [62].

In a study by Vaes et al. [63], a strong positive correlation between the change in 25(OH)D and 24,25(OH)_2_D was found after supplementation. Serum 24,25(OH)_2_D showed a dose–response relationship that was parallel to serum 25(OH)D patterns, suggesting that this metabolite is an index of vitamin D deficiency and, indirectly, stimulates the catabolic pathway to regulate 1,25(OH)_2_D [64]. This produces the hypothesis that knowledge of 24,25(OH)_2_D levels may allow for the customization of the dose required to reach the target level of 25(OH)D [64,65].

Until now, only a limited number of in vivo studies have evaluated the efficacy of calcifediol compared with oral cholecalciferol in increasing serum 25(OH)D concentrations. Stamp et al. [66] demonstrated for the first time in the early 1970s that calcifediol seems to be about 10-fold more potent than cholecalciferol/ergocalciferol in increasing serum 25(OH)D concentrations. However, the study had major limitations, such as the lack of homogeneity of the groups, the inclusion of subjects with metabolic bone diseases, the administration of either cholecalciferol or ergocalciferol in the vitamin D arm without further sub-analysis, and differences in terms of treatment duration between groups.

As far as we know, four randomized double-blind controlled trials, three randomized open-label trials, and one prospective open-label trial have aimed to compare the ability of calcifediol with that of cholecalciferol to increase serum 25(OH)D: three studies used a single dosage of the investigational product and the others used multiple dosages (Table 1).

To summarize, these studies have shown that calcifediol seems to be more powerful than cholecalciferol (2–6 times), and that its use results in a quicker increase of 25(OH)D.

### 4.2. Randomized Double-Blind Controlled Trials

#### 4.2.1. Bischoff-Ferrari et al.

Bischoff-Ferrari et al. [73] designed one of the first randomized double-blind controlled trials that aimed to compare a single dose of calcifediol with that of cholecalciferol. In this study, 20 healthy postmenopausal women with an average 25(OH)D level of 13.2 ng/mL and a mean age of 61.5 years were randomized to 20 μg calcifediol or 20 μg (800 IU) cholecalciferol daily for four months. The authors demonstrated an immediate and sustained increase in 25(OH)D serum levels in the calcifediol group compared with the cholecalciferol group, and determined that a slow increase in 25(OH)D levels occurred. Calcifediol demonstrated a decrease of 5.7 mmHg in systolic blood pressure.

#### 4.2.2. Cashman et al.

In a 10 week randomized, double-blind, placebo-controlled trial, Cashman et al. [72] compared the potency of multiple dosages of oral calcifediol (7 or 20 μg/day) versus cholecalciferol (daily dose of 20 μg). In this study, 56 overweight healthy subjects (average body mass index (BMI) = 28.3 kg/m^2^) with an average serum 25(OH)D level of 17.5 ng/mL were included. At weeks 5 and 10, the increase in serum 25(OH)D achieved with 20 μg calcifediol daily was significantly higher than that in the groups that used either 20 μg cholecalciferol or 7 μg calcifediol daily or a placebo. Only calcifediol (both dosages) showed a significant decrease in serum PTH concentrations at weeks 5 and 10 when compared with the placebo. The authors highlighted that oral calcifediol seemed to be 4.2–5 times more potent than oral cholecalciferol.

#### 4.2.3. Jetter et al.

In 2014, Jetter et al. [71] published a seven-arm, randomized, double-blind, controlled parallel-group study using multiple dosages of calcifediol and cholecalciferol. In this trial, 35 healthy postmenopausal women aged 50–70 years with a mean baseline BMI between 18 and 29 kg/m^2^ and serum 25(OH)D of about 13 ng/mL were randomized to a 15 week trial of 20 μg calcifediol or cholecalciferol daily, a 15 week trial of 140 μg calcifediol or cholecalciferol weekly, or a single bolus of either 140 μg calcifediol or cholecalciferol or both. Calcifediol seemed to be about two to three times more potent in increasing plasma 25(OH)D concentrations than cholecalciferol for the daily dosages, and five to six times more potent when weekly dosages were compared. Plasma 25(OH)D concentrations of 30 ng/mL were reached more rapidly and reliably with calcifediol. One of the important limitations of this study was the non-homogeneous period of randomization and follow-up (from January to July).

#### 4.2.4. Vaes et al.

Vaes et al. [63] compared the efficacy of multiple dosages of calcifediol and cholecalciferol in a double-blind randomized controlled trial (RCT) that included 59 men and women aged ≥65 years with a baseline 25(OH)D of 15.8 ± 4.8 ng/mL. The study population was randomized to 5, 10, or 15 μg calcifediol or 20 μg cholecalciferol per day for a period of 24 weeks. Patients treated with cholecalciferol demonstrated increased 25(OH)D levels as high as 28 ng/mL within 16 weeks, whereas subjects treated with 10 or 15 μg of calcifediol had increased 25(OH)D levels to >30 ng/mL in 8 and 4 weeks, respectively. The low dose of oral calcifediol (5 μg) had a relative potency of about 1.04 versus cholecalciferol, whereas both the other dosages (10 and 15 μg) demonstrated a relative potency of 3 and 2.8, respectively. In this study, dietary vitamin D and calcium intake were recorded only at the baseline, using a food frequency questionnaire.

### 4.3. Open-Label Trials

#### 4.3.1. Barger-Lux et al.

A prospective open-label study that compared multiple dosages of cholecalciferol with multiple dosages of calcifediol was published in 1998 by Barger-Lux et al. [70]. The authors assigned 116 healthy adults with a mean age of 28 years to nine groups using three dosages of cholecalciferol (25, 250, or 1250 μg/day for eight weeks), three dosages of calcifediol (10, 20, or 50 μg/day for four weeks), or three dosages of calcitriol (0.5, 1.0, or 2.0 μg/day for two weeks). The relative potency of calcifediol in increasing 25(OH)D levels was 3.3–3.5 times higher than that of cholecalciferol when comparing a low dosage of cholecalciferol (25 μg) with similar dosages of oral calcifediol (10, 20, or 50 μg/day). When the authors compared high dosages of cholecalciferol (10–50,000 IU/day) with the highest dose of calcifediol (50 μg or 2000 IU/day), oral calcifediol was shown to be seven- to eight-fold more potent than cholecalciferol.

#### 4.3.2. Rossini et al.

In 2005, Rossini et al. [69] evaluated the potency of a single dosage of calcifediol and cholecalciferol in restoring 25(OH)D concentrations in a randomized, open-label controlled trial. In this study, 271 women affected by postmenopausal osteopenia or osteoporosis complicated by hypovitaminosis D (the mean value of 25(OH)D was 8.8 ng/mL) were randomized to consume either a weekly orally administered treatment of 20 drops (4000 IU) of calcifediol plus daily calcium supplementation, or a 800–880 IU daily dose of cholecalciferol in combination with 1 g calcium. The calculated relative potency of calcifediol versus cholecalciferol in increasing 25(OH)D was 1.66.

#### 4.3.3. Navarro-Valverde et al.

Another RCT, conducted by Navarro-Valverde et al. [68], compared the efficacy of a single dosage of cholecalciferol (20 μg/day) versus multiple dosages of calcifediol (20 μg/day, 266 μg/week, or 266 μg every other week). For one year, the investigators followed 40 osteopenic postmenopausal women with an average age of 67 years and a mean 25(OH)D level of 15 ng/mL. The authors demonstrated that the metabolites were not equipotent, highlighting that calcifediol acted faster and was three to six times more potent in obtaining serum levels of 25(OH)D in the medium- to long-term when compared with cholecalciferol.

#### 4.3.4. Shieh et al.

Shieh et al. [67] assessed the effects of a single dosage of cholecalciferol and calcifediol on total and free 25(OH)D levels and PTH changes. The authors studied 35 multiethnic healthy adults ≥18 years of age with 25(OH)D levels of ≤20 ng/mL. Subjects received 60 μg (2400 IU) of cholecalciferol or 20 μg of calcifediol daily for 16 weeks. In the calcifediol group, the mean total 25(OH)D increased to ≥30 ng/mL within four weeks. Among those who received cholecalciferol, the mean total 25(OH)D remained at <30 ng/mL for the entire study. At the end of the study, total and free 25(OH)D levels had increased to a greater extent with calcifediol than with cholecalciferol. The authors found that higher levels of both total and free 25(OH)D were significantly associated with a future decline in serum PTH.

## 5. Calcifediol in Different Clinical Settings

Due to the chemical–physical diversity with respect to cholecalciferol, particularly its polarity and different degrees of lipophilicity, we assess in this section the specific features of this metabolite under conditions such as malabsorption and obesity, in addition to SHPT and an increased risk of falls.

### 5.1. Calcifediol in SHPT

In subjects with SHPT due to vitamin D deficiency, supplementation with appropriate doses of calcifediol, cholecalciferol, or ergocalciferol was able to normalize the levels of 25(OH)D and PTH [74,75,76], although the evidence on the use of calcifediol was limited.

The correction of 25(OH)D deficiency by nutritional vitamin D supplementation is suggested by KDIGO (Kidney Disease—Improving Global Outcome) guidelines to prevent and treat subclinical hyperparathyroidism in chronic kidney disease (CKD) [77]. The safer pharmacokinetics of the modified-release (MR) formulation of calcifediol were able to replenish 25(OH)D levels with minimal impact on vitamin D catabolism and fibroblast growth factor-23 activation [78].

A double-blind, placebo-controlled trial indicated that oral MR calcifediol administered in different doses is safe and highly effective in increasing total 25(OH)D serum concentrations to ≥30 ng/mL and reducing plasma PTH concentrations in adult CKD patients [79]. Newer studies are needed to demonstrate the efficacy of calcifediol in patients with CKD and SHPT [80].

### 5.2. Calcifediol and Increased Risk of Falls

In a one year, double-blind, randomized clinical trial on 200 community-dwelling men and women who were 70 years and older with a prior fall, monthly treatments were administered to three distinct study groups: a low-dose control group receiving 24,000 IU of vitamin D_3_, a group receiving 60,000 IU of vitamin D_3_, and a group receiving 24,000 IU of vitamin D_3_ plus 300 μg of calcifediol. The primary endpoint was improved lower extremity function and the achievement of 25(OH)D levels of at least 30 ng/mL at 6 and 12 months. A secondary endpoint was monthly reported falls. The incidence of falls differed significantly among the treatment groups, with higher incidences in the 60,000 IU group (66.9%; 95% confidence interval (CI): 54.4–77.5%) and the 24,000 IU plus calcifediol group (66.1%; 95% CI: 53.5–76.8%), compared with the 24,000 IU group (47.9%; 95% CI: 35.8–60.3%) (*p* = 0.048). Serum levels of 25(OH)D in the highest quartile for all participants ranged from 44.7 to 98.9 ng/mL, with the probability of falling being 5.52 times higher in the highest quartile (95% CI: 2.1–14.5) than that in the lowest quartile (serum levels of 25(OH)D of 21–30 ng/mL). Thus, although higher monthly doses of vitamin D were effective in reaching a threshold of at least 30 ng/mL of 25(OH)D, they had no benefit on lower extremity function and were associated with an increased risk of falls compared with the 24,000 IU group [81].

### 5.3. Calcifediol and Malabsorption

The differences in polarity between vitamin D_3_ and 25(OH)D can affect the mechanism of absorption of the two forms. It has been shown that 25(OH)D is absorbed more proximally in the small bowel and was not as dependent on bile and micelle formation for its absorption as was vitamin D [82].

Malabsorption of cholecalciferol and, to a lesser extent, calcifediol, occurs after gastrectomy, in celiac diseases, and in other small bowel diseases [58,83]. Several consensuses and guidelines advise the use of calcifediol in these scenarios [62,84,85].

### 5.4. Calcifediol and Obesity

Either vitamin D deficiency or its suboptimal status, 25(OH)D <20–40 ng/mL, have been associated with defective metabolic phenotypes, such as insulin resistance, type 2 diabetes, and cardiovascular diseases. All of these conditions are also commonly linked with being overweight and obese [86,87]. A poor vitamin D status in the morbidly obese is frequently reported, and a graded relationship between vitamin D status and BMI has been described in the general population, probably due to vitamin D sequestration in adipose tissue, volumetric dilution, or negative feedback mechanisms from increased circulating 1,25(OH)_2_D [88].

In an animal study, by assessing ex vivo activity of isolated livers from obese mice, the expression of *CYP2R1* was reported to be significantly reduced in obese versus lean mice, a finding that may account in part for the observed decrease in circulating 25(OH)D [22].

Calcifediol may be more effective than cholecalciferol in obese patients [89,90]. In a prospective randomized study on 54 normal weight and 67 obese men assigned to supplementation with 50 µg/week of calcifediol or 150 µg/week of vitamin D_3_ for one year, supplementation with calcifediol was found to be more effective in achieving vitamin D sufficiency in obese subjects, but not in normal weight subjects. This finding is probably the result of a reduction in the catecholamine-induced release of D_3_ and 25(OH)D and the altered activity of vitamin D-metabolizing enzymes in adipose tissue, defined as dysfunctional in obese subjects [90].

Bariatric surgery, and particularly malabsorptive procedures such as biliopancreatic diversion, is a well-known cause of severe vitamin D deficiency. Data have shown the persistence of hypovitaminosis D despite substantial weight loss following surgery [91]. Some authors have demonstrated that women who had been submitted to bariatric surgery at least three years before presented lower 25(OH)D values when compared with normal paired controls: 77.1% of the surgical patients presented vitamin D insufficiency/deficiency and 41.7% presented SHPT [92].

The ES [93], the American Association of Clinical Endocrinologists (AACE), the Obesity Society (TOS), the American Society for Metabolic & Bariatric Surgery (ASBMS) [94], and the Interdisciplinary European Guidelines [95] have all recommended vitamin D supplementation for the post-operative care of bariatric surgery patients. The ES recommends 50,000 IU vitamin D one to three times weekly [93]. The AACE, TOS, and ASBMS guidelines recommend 3000–6000 IU of vitamin D daily [94].

Currently, we do not have evidence-based guidelines that support the preferential use of calcifediol after malabsorptive bariatric surgery, but the little evidence that does exist seems to support this assumption [96].

### 5.5. Drugs Interfering with Calcifediol Metabolism

Many drugs that interact with the pregnane X receptor (PXR) stimulate the expression of 24-hydroxylase, which increases the degradation of 25(OH)D, reducing the circulating levels and leading to a vitamin D deficiency. Paradigmatic examples of such drugs are the anticonvulsant inductors of cytochrome P-450 (carbamazepine, phenobarbital, and diphenylhydantoin) [97,98,99,100], but other drugs can also bind to the PXR and interfere with the metabolism of vitamin D (glucocorticoids, antineoplastics, and antiretrovirals) [98].

Nevertheless, some studies described vitamin D deficiency in subjects affected by bipolar disorder, schizophrenia, schizoaffective disorder or other psychotic disorders, and evidence suggests that vitamin D could have a pathophysiological role [101,102]. However, only one study has evaluated vitamin D administration in subjects with chronic schizophrenia, demonstrating that supplementation did not affect psychosis, mood, or metabolic status [103]. Furthermore, it has been demonstrated that vitamin D supplementation in drug resistant epilepsy patients does not result in a reduction of seizure frequency [104].

Supplementation with native vitamin D can improve the bone turnover indices and levels of PTH in these kinds of subjects [105]. In these cases, the ES guidelines suggest dosages of vitamin D_3_ that are two to three times higher than the usual dose [3], and others suggest doses of >1800 IU/day [105]. Potential benefits of calcifediol supplementation in improving physical and mental health in these subjects should be explored.

## 6. Conclusions: Time to Consider Calcifediol as an Adequate Supplement of Vitamin D?

Data from the literature are in accord in recommending vitamin D supplementation for all subjects at risk of vitamin D deficiency (Table 2), and the determination of vitamin D deficiency is cost-effective. 25(OH)D values of <20 ng/mL indicate a deficiency, but the requirement of supplementation is still controversial for values between 20 and 30 ng/mL [85]. The majority of the data on vitamin D supplementation in relation to human health almost exclusively involve cholecalciferol in registered clinical trials on anti-fracture drugs in subjects with reduced bone mass; the data highlight skeletal-related endpoints. All these trials have administered cholecalciferol supplementation, although sometimes with different dosages. Consequently, at the moment, robust, evidence-based data that unequivocally demonstrate the effects of the vitamin D hydroxylated metabolite on skeletal health are not available.

In general, in subjects with hypovitaminosis D, oral calcifediol supplementation is more powerful for increasing serum 25(OH)D concentrations than cholecalciferol. In particular, the calculated relative potency of calcifediol versus cholecalciferol varied between 1.7 and 8, depending on the doses, different pharmacokinetics, and basal 25(OH)D serum levels [6]. A formula has been proposed for calculating the expected increase in circulating 25(OH)D levels when vitamin D is supplemented, which considers age, the baseline 25(OH)D status, and body weight [106]. With this approach, much higher doses of vitamin D_3_ would be required to realize the same increase in serum 25(OH)D concentration as that observed with calcifediol [107]. In patients affected by hypovitaminosis D without reduced bone mass, calcifediol can be taken instead of cholecalciferol, because it is better able to increase circulating 25(OH)D levels without the need to use micro- or macro-boli.

To summarize, compared with cholecalciferol, calcifediol is absorbed the best, has a different volume of distribution, is independent from hepatic 25-hydroxylase, and produces a more rapid increase in circulating levels of 25(OH)D [6,62]. In particular, calcifediol is a more polar and soluble metabolite that may display smaller volumes of distribution, and less trapping by the adipose tissue [62]. Consequently, these aspects affect both its intestinal absorption and circulating DBP transport, as well as the whole body distribution of its orally administered metabolite, displaying a much shorter half-life (approximately 10–13 days) than the parental cholecalciferol [16,58,108,109,110]. The use of calcifediol might be more cost-effective in obese patients and in those with malabsorption syndromes. Its use may be beneficial in patients taking drugs that interfere with the hepatic cytochrome P-450 enzyme system, particularly corticosteroid drugs or anticonvulsants. Thus, at least in these specific conditions, calcifediol can be used as an alternative to cholecalciferol [111] and, assuming the above-described higher pharmacologic activity of oral calcifediol relative to oral cholecalciferol [6], the cost per IU of calcifediol could be about six time lower than that of cholecalciferol (according to the market prices in Italy in 2019) [112,113], although such a direct comparison between the costs of these different molecules cannot lead to an accurate and adequate proposal.

Quesada-Gomez JM and colleague suggested “oral calcifediol as a valid and favorable alternative for the prevention or treatment of osteoporosis” [6]. Instead, we are strongly convinced that further studies are needed to investigate the efficacy and safety of calcifediol in the clinical setting of bone fragility before suggesting its use as a safe and efficient alternative vitamin D supplement to be routinely used.

## Figures and Tables

**Table 1:** Studies focused on the comparison between calcifediol and cholecalciferol.

Authors	Shieh [67]	Navarro-Valverde [68]	Rossini [69]	Barger-Lux [70]	Vaes [63]	Jetter [71]	Cashman [72]	Bischoff-Ferrari [73]
Type of study	Open-label RCT	Open-label RCT	Open-label RCT	Prospective open-label study	Double-blind RCT	Double-blind RCT	Double-blind, placebo-controlled RCT	Double-blind RCT
Study population	35 multiethnic healthy adults ≥18 years of age with a mean BMI of 26.5 kg/m^2^	40 postmenopausal osteopenic women with an average age of 67 years and a BMI of 26.4 ± 4 kg/m^2^	271 postmenopausal osteopenic or osteoporotic women	116 healthy adults with a mean age of 28 years	59 subjects: men and women aged >65 years with a BMI between 20 and 35 kg/m^2^.	35 healthy women aged 50–70 years with a baseline BMI between 18 and 29 kg/m^2^	56 healthy adults aged ≥50 years with a mean BMI of 28.3 ± 4.8 kg/m^2^	20 healthy postmenopausal women with a mean age of 61.5 ± 7.2 years and a BMI between 18 and 29 kg/m^2^
Baseline Mean 25(OH)D level Assay	16.6 ± 3.1 ng/mL chemiluminescence immunoassay	37.5 ± 10 nmol/L HPLC and ultraviolet detection method	22 nmol/L radioimmunoassay	67 ± 25 nmol/L competitive protein-binding assay with chromatography	39.4 ± 11.9 nmol/L IDXLC-MS/MS	12.54 ± 3.51 ng/mL HPLC–MS/MS	43.6 ± 12.3 nmol/L ELISA	13.2 ± 3.9 ng/mL HPLC–MS/MS
Intervention	60 μg chol/day 20 μg calcif/day for 16 weeks	20 μg chol/day, 20 μg calcif/day, 266 μg calcif/week, 266 μg calcif/every other week for one year	4000 IU calcif/week, 800–880 IU chol/day for one year	25 μg chol/day, 250 μg chol/day, 1250 μg chol/day for 8 weeks; 10 μg calcif/day, 20 μg calcif/day, 50 μg calcif/day for 4 weeks 0.5 μg calcit/day, 1.0 μg calcit/day, 1.5 μg calcit/day for 2 weeks.	20 μg chol/day; 5 μg calcif/day, 10 μg calcif/day, 15 μg calcif/day for 24 weeks	20 μg chol/day; 20 μg calcif/day, 140 μg calcif/week, 140 μg chol/week, for 15 weeks, bolus 140 μg calcif, bolus 140 μg chol both bolus (chol and calcif)	placebo, 20 μg chol/day, 7 μg calcif/day, 20 μg calcif/day, for 10 weeks	20 μg chol/day; 20 μg calcif/day, for 4 months
Results	The mean total 25(OH)D significantly increased to ≥30 ng/mL by 4 weeks of calcif, while among the chol group, the mean total 25(OH)D remained <30 ng/mL for the entire study.	Calcif increased to significantly higher 25(OH)D serum levels compared with daily chol. The increase in 25(OH)D serum levels was almost 2 times higher in the group treated with weekly calcif.	The compliance with the weekly calcif was over 90%, and determined serum levels of 25(OH)D were similar to those obtained with chol daily.	Treatment with calcif significantly increased 25(OH)D serum levels more than chol.	Calcif significantly elevated serum 25(OH)D concentrations more rapidly compared with chol.	20 μg calcif given daily or 140 μg given weekly appeared to significantly correct vitamin D deficiency more rapidly and reliably than the same dose of daily or weekly chol.	20 μg calcif daily significantly increased 25(OH)D serum levels more than either 20 μg chol or 7 μg calcif daily.	Immediate sustained and significant increase in 25(OH)D serum levels with calcif.
Notes	After 12 months, calcif was between 3 and 5 times more potent than chol.	Calcif was 3–6 times more potent in increasing 25(OH) D serum levels compared with chol.	The potency of calcif versus chol in increasing 25 (OH)D was 1.66. This study aimed to evaluate the compliance of patients and not the efficacy.	The potency in increasing 25(OH)D levels of calcif was 3.3–3.5 times more than chol at a low dosage and 7–8 times more for the highest dosages of both products.	5 μg of calcif had a potency of about 1.04 versus chol, whereas for both other dosages (10–15 μg), the potency was about 3.	Daily calcif was 2–3 times more potent than chol, and weekly calcif was 5–6 times more potent than chol.	Calcif seemed to be 4.2–5 times more potent than chol. Both dosages of calcif reduced serum PTH vs. placebo.	Potency of calcif vs. chol was 3.4. Calcif demonstrated a decrease of 5.7 mmHg SBP.

BMI: body mass index; calcif: calcifediol; calcit: calcitriol; chol: cholecalciferol; ELISA: enzyme-linked immunosorbent assay; IDXLC-MS/MS: isotope dilution-online solid phase extraction liquid chromatography-tandem mass spectrometry; HPLC-MS/MS: liquid chromatography coupled to tandem mass spectrometry detection; SBP: systolic blood pressure; IU: international unit; RCT: randomized clinical trial.

**Table 2:** Categories of patients that should be screened for vitamin D deficiency.

Osteomalacia
Osteoporosis (particularly if bone-active drugs are to be used)
Older adults with a history of falls
Older adults with a history of non-traumatic fractures
Pregnant and lactating women
Obese children and adults
People with insufficient sun exposure
Malabsorption syndromes (congenital or acquired) and bariatric surgery
Chronic kidney disease
Hepatic failure
Cystic fibrosis
Hyperparathyroidism
People taking drugs that interfere with vitamin D metabolism (antiseizure medications, glucocorticoids, AIDS medications, antifungals, cholestyramine)
Granulomatous disorders and some lymphomas
